# Perspectives of the Application of Non-Steroidal Anti-Inflammatory Drugs in Cancer Therapy: Attempts to Overcome Their Unfavorable Side Effects

**DOI:** 10.3390/cancers15020475

**Published:** 2023-01-12

**Authors:** Vaikunthavasan Thiruchenthooran, Elena Sánchez-López, Anna Gliszczyńska

**Affiliations:** 1 Department of Food Chemistry and Biocatalysis, Wrocław University of Environmental and Life Sciences, Norwida 25, 50-375 Wrocław, Poland; 2 Department of Pharmacy, Pharmaceutical Technology and Physical Chemistry, University of Barcelona, 08028 Barcelona, Spain; 3 Institute of Nanoscience and Nanotechnology (IN2UB), University of Barcelona, 08028 Barcelona, Spain; 4 Unit of Synthesis and Biomedical Applications of Peptides, IQAC-CSIC, 08034 Barcelona, Spain

**Keywords:** NSAIDs, chemotherapeutic drugs, phosphatidylcholine, terpenoids, anti-cancer therapy

## Abstract

**Simple Summary:**

The application of non-steroidal anti-inflammatory drugs (NSAIDs) in cancer therapy has been a widely studied topic for almost four decades. This review summarizes the information about the role of chronic inflammation in the process of carcinogenesis and the evidence regarding the anti-cancer activity of the most frequently used NSAIDs. Despite the promising results of NSAIDs, the possibilities of the practical application of this group of drugs in chemoprevention and cancer therapies on a clinical basis are still impossible due to individual side effects. The main concern is their gastrointestinal toxicity. For this reason, several strategies are still under investigation to reduce toxicity and improve the efficacy of NSAIDs. In this review, we emphasize the effectiveness of combinations of NSAIDs with chemotherapeutic drugs in in vitro and in vivo models as well as in clinical trials. We also present new concepts based on the molecular hybridization of anti-inflammatory drugs with other biologically active molecules, such as phospholipids and terpenes, as solutions for reducing NSAID side effects. The production of derivatives with enhanced biological potentials could also be carried out to target rare types of cancers. Therefore, this manuscript provides new insight into the possibilities of re-purposing NSAIDs for new, outside-the-current-scope applications.

**Abstract:**

Non-steroidal anti-inflammatory drugs (NSAIDs) express anti-tumoral activity mainly by blocking cyclooxygenase-2 involved in the synthesis of prostaglandins. Therefore, in the last few decades, many have attempted to explore the possibilities of applying this group of drugs as effective agents for the inhibition of neoplastic processes. This review summarizes the evidence presented in the literature regarding the anti-tumoral actions of NSAIDs used as monotherapies as well as in combination with conventional chemotherapeutics and natural products. In several clinical trials, it was proven that combinations of NSAIDs and chemotherapeutic drugs (CTDs) were able to obtain suitable results. The combination with phospholipids may resolve the adverse effects of NSAIDs and deliver derivatives with increased antitumor activity, whereas hybrids with terpenoids exhibit superior activity against their parent drugs or physical mixtures. Therefore, the application of NSAIDs in cancer therapy seems to be still an open chapter and requires deep and careful evaluation. The literature’s data indicate the possibilities of re-purposing anti-inflammatory drugs currently approved for cancer treatments.

## 1. Introduction

Cancer, along with diabetes and cardiovascular and respiratory diseases, belongs to the top four most common non-communicable diseases in the world. The global cancer mortality rate was almost 10 million in 2020, whereas the number of newly diagnosed cases reached 19.3 million [1]. Moreover, the mortality rate is expected to rise by 47% in 2040 [1]. According to the WHO’s reports from 2020, the most common types of cancer-causing mortality were lung (1.8 million), colon and rectum (0.94 million), liver (0.83 million), stomach (0.77 million) and breast (0.69 million) cancers [1].

A strong relationship between the occurrence of inflammatory processes and cancer incidence has been highlighted since the mid-19th century. In 1863, Rudolf Virchow put forward the hypothesis that the lymphoreticular infiltrates of immune cells during chronic inflammation constitute the main cause of cancer [2]. Then, in 1986 Dvorak reported histological evidence that inflammation and cancer progression share developmental similarities [3]. On this basis, it was concluded that targeting chronic inflammation with the use of non-steroidal anti-inflammatory drugs (NSAIDs) may constitute a valuable strategy in the fight against cancer. NSAIDs have a long history of certified abilities as antipyretic, analgesic, and anti-inflammatory agents [4]. For instance, it was proven that acetylsalicylic acid monthly oral administration could prevent approximately 37 cardiovascular events, such as myocardial infarction, thrombotic stroke, and death among 1000 patients [5]. The first evidence of the possibility to re-purpose NSAIDs appeared in the early 1980s, following successful animal research. Experiments conducted by Pollard and Lucket on rats administered with indomethacin for several weeks confirmed the protective effect of this drug against induced colorectal cancer by reducing the number and size of tumors [6]. Similar effects were observed in experiments with sulindac, ibuprofen, and aspirin [7,8]. In the case of sulindac, it was also confirmed that the drug’s suppressive effect was observed in both the invasive and non-invasive tumoral forms [7]. Animal studies have indicated that the inhibitory effect of NSAIDs is tumor-stage-dependent. Sulindac inhibited the development of colon cancer in rats only when the drug was administered throughout the experimental period since the time of tumor induction [9], while for piroxicam, no such relationship was found [10]. After that, a series of clinical trials were started on the possibilities of extending the use of NSAIDs in anti-cancer therapeutics.

The Food and Drug Administration (FDA) only approves a few de novo drugs, which are released on the market annually. In 2018, 2019, and 2020 the numbers of de novo drugs approved were 59, 48, and 53, respectively, while the European Medicines Agency (EMA) approved only 30 in 2019 and 39 new drugs in 2020 [11,12,13]. In view of the scale of the problem that cancer is today and the time needed to discover and approve drugs that are effective in their chemoprevention and therapy, the best strategy seems to be to re-purpose the drugs already approved to treat other diseases. In this aspect, NSAIDs and their combinations and conjugation with natural products, such as phospholipids and terpenoids, have great potential and importance, which are presented in the following parts of this review.

## 2. Anti-Inflammatory Activity of Non-Steroidal Anti-Inflammatory Drugs

**Figure 1:**
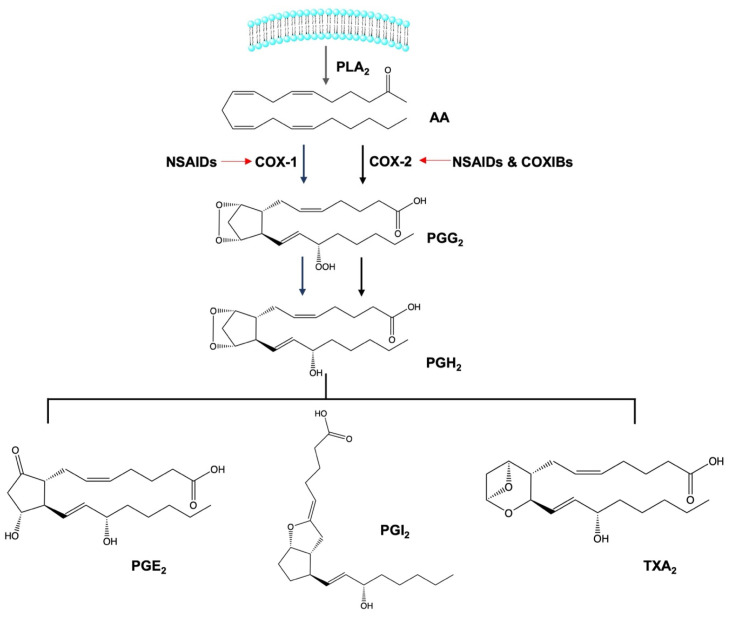
Effect of NSAIDs on PG biosynthesis.

Concepts about the mechanisms of action of NSAIDs were introduced two decades ago [14], and the results of the studies supported the ability of NSAIDs to prevent inflammation mainly by inhibiting prostaglandin (PG) biosynthesis. The effect of NSAIDs on PG synthesis is shown in Figure 1. The first response to pro-inflammatory cytokines and chemokines is that arachidonic acid (AA) from the cellular phospholipid bilayer is oxidized to prostaglandin G2 (PGG2), and peroxide is reduced to prostaglandin H_2_ (PGH_2_) by the cyclooxygenase (COX) enzyme, also known as prostaglandin endoperoxide H synthase (PGH synthase). In the transformation of AA to PGH_2_, COX acts as a deoxygenase and peroxidase.

The conversion of PGG_2_ to unstable PGH_2_ is known to be the key regulatory step of this pathway. Further, by specific enzymes in different tissues, PGH_2_ is metabolized to different PGs, inclusively PGE_2_, PGI_2_, and thromboxane A_2_ (TXA_2_) [15]. Moreover, there are two COX isoforms involved in this pathway, namely COX-1 and COX-2. Additionally, the COX-3 isoform has also been discovered but has no reported involvement in PG synthesis [14].

Both COX-1 and COX-2 produce PGs and are expressed by two different genes located in chromosome 9 and chromosome 1, respectively [16]. COX-1 is ubiquitously expressed in many tissues and cells and produces PGs constantly and equally in normal and pathological conditions. Functionally, COX-1-linked PGs are associated with renal function, gastric mucosal maintenance, the stimulation of platelet aggregation, and vasoconstriction. COX-2-linked PGs are helpful to organs such as the kidney, female reproductive organs, brain, and bones [16]. In the kidney, COX-2-linked PGs signal through the peroxisome proliferator-activated receptor-β/δ (PPARβ/δ) to control renal blood flow [17]. In the corpus luteum, COX-2 is associated with ovulation, implantation, and decidualization, and in the brain is associated with memory consolidation, synaptic activity, and functional hyperemia, also being crucial for bone fracture healing [16]. COX-2 is linked to inflammation and was reported to be elevated in human melanoma models [18]. Its pathological significance is under consideration in NSAID applications. COX-2 constitutes the immediate response to pro-inflammatory cytokines and mediators, tumor promoters, DNA damaging agents, growth factors, and oxidizing agents, and the derived PGE_2_ is found to be pro-carcinogenic. It is associated with melanoma cell proliferation, metastasis, and angiogenesis, and exerts its potential by binding to the E series of prostaglandin receptors 1–4 (EP 1–4) [15].

Considering the important role of COX-1-derived PGs and COX-2/PGs in pro-inflammatory promotion, poor COX-1 inhibition improves COX-2-specific inhibition and may be a suitable approach for cancer therapeutics. There are a number of similarities between COX-1 and COX-2, such as they both share similar molecular weights (70 kDa and 72 kDa) and a similar number of amino acids (602 and 604), possessing 60% homology at the amino acid level [16,19]. However, the positional differences of the amino acids distinguish the structural features, such as the large hydrophobic binding pocket, which occurs in COX-2 but not in COX-1 because of the differences in the valine in COX-2 at positions 89 and 523 instead of isoleucine in COX-1 [16,20]. Functionally COX-2 accepts a wider range of fatty acids as substrates than COX-1 [20]. The amino acid differences further help to omit COX-1 inhibition, such as meloxicam acting on COX-2, avoiding COX-1 inhibition. This selectivity is due to a single amino acid difference in the same position at the hydrophobic COX channel containing isoleucine in COX-1 and valine in COX-2 at position 509 [21]. This approach led to the further development of selective COX-2 inhibitors, named COXIBs. For example, the first well-known COXIB, celecoxib, was able to inhibit COX-2, avoiding COX-1 inhibition. In a recent clinical paper, Bonnesen and Schmidt reported that NSAIDs should be divided into COX-1 inhibitors, non-selective NSAIDs, older COX-2 inhibitors, and newer COX-2 inhibitors (COXIBs) [22]. In their report, the COX-1 inhibitors included flurbiprofen, ketoprofen, fenoprofen, oxaprozin, and tolmetin and the non-selective dose-dependent inhibitors consisted of indomethacin, ibuprofen, naproxen, piroxicam, ketorolac, and nabumetone. The older COX-2 inhibitors included sulindac, meloxicam, salsalate, etodolac, mefenamic acid, diclofenac, and nimesulide. Further, the COX-2 inhibitory IC_50_ concentrations of the COXIBs, in descending order, included celecoxib, valdecoxib, rofecoxib, etoricoxib, and lumiracoxib [22]. Bonnesen and Schmidt proposed this requirement of the subdivisions of the NSAIDs, and this requires future research to reach a wider acceptance. Thus, throughout this review, we used the terms COX-1 selective inhibitors, non-selective COX inhibitors, and selective COX-2 inhibitors.

## 3. Evidence of the Anti-Cancer Mechanism of Action of NSAIDs toward Tumoral Cell Lines

Among the NSAIDs, non-COX selective and COX-2 dependent inhibitors are able to exert anti-tumoral activities by inhibiting the COX-2 enzyme. For instance, indomethacin suppresses the proliferation of human pancreatic stellate cells by inhibiting COX-2 activity [23]. In addition, celecoxib inhibits the proliferation, migration, and invasion of human hepatocellular carcinoma BEL-7402 cells primarily by inhibiting COX-2 expression [24]. However, there is considerable evidence for the anti-tumoral activities of NSAIDs beyond COX-2 inhibition. For instance, SC-560, a non-COX-2 inhibitor, suppresses the colonization of the human hepatocellular carcinoma HuH-6 cell line [25]. In addition, the results reported by Hurst et al. confirmed that mavacoxib is able to induce apoptosis and inhibits the migration of cancer cells independently of elevated COX-2 expression levels. This suggests that NSAIDs may play roles as anti-cancer agents independently of tumor COX-2 expression [26].

Intracellular tumor cell signaling is a complex cascade of connections that allow tumoral cells to survive, proliferate, and migrate. NSAIDs are able to induce the apoptosis of cancer cells also through the downregulation of B-cell lymphoma-2 (Bcl-2), an anti-apoptotic protein that regulates mitochondrial thiol reduction and further regulates the mitochondrial permeability of apoptotic proteins such as apoptotic protease activating factor 1 (Apaf-1). The downregulation of anti-apoptotic proteins promotes tumor cell apoptosis. In gastric cancer cells (BGC-823), celecoxib inhibits the expression of two anti-apoptotic proteins, Bcl-2 and Fas ligand (FasL), and increases the expression of the apoptotic Fas protein [27]. In another study, it was proven that ibuprofen inhibits Bcl-2 transcription and stimulates the transcription of an apoptosis regulator protein called Bcl-2 associated X (Bax) in an adenocarcinoma gastric AGS cell line [28].

Another aspect is NSAIDs’ ability to downregulate the tumoral cell cycle and survival. The cell cycle and survival of tumor cells depend on phosphoinositol 3-kinase (PI3K)/protein kinase B (Akt) signaling. Thus, the cause of a misregulated cell cycle is unknown in Akt hyperactivation, although it is known that the Akt signaling pathway induces cyclin A activation and promotes cell cycle progression [29]. Thus, in recent years, NSAIDs were intensely evaluated to identify which ones exhibited activity that downregulated the cell cycle and cell proliferation by downregulating PI3K/ Akt signaling. Collectively, several NSAIDs undergo inhibition via PI3K/ Akt signaling. Naproxen downregulated P13K/Akt signaling by directly binding to P13K molecules to arrest the cells at the G_0_–G_1_ phase in the studies carried out in human bladder cancer cell lines (UM-UC-5 and UM-UC-14) [30]. Furthermore, celecoxib inhibits Akt and cyclin D1 expression in head and neck squamous cell carcinoma cell lines (HN30 and HN31) [31]. In addition, diclofenac dephosphorylated Akt during the apoptosis of human colon cancer cells (HCT116) [32]. This supports the hypothesis that the downregulation of P13K/Akt signaling caused by NSAIDs not only inhibits the cell cycle but also causes apoptosis.

Another crucial mechanism of NSAIDs’ anti-cancer activity is the Wnt/β-catenin signaling pathway. This pathway is an important cascade for cancer cell renewal and migration [33]. For instance, acetylsalicylic acid downregulates the migration and cell viability of a human colon cancer cell line (SW480) by inhibiting Wnt signaling [34]. Furthermore, NSAIDs upregulate and downregulate the genes associated with tumorigenesis. In AGS cells, celecoxib suppressed tumor cell survival through the upregulation of wild-type p53 gene expression [28]. Furthermore, diclofenac downregulated c-MYC (cellular–myelocytomatosis) gene expression and inhibited leukemia cell proliferation (U937) [35]. In addition, acetylsalicylic acid reduced the level of nuclear factor-kB (NFkB) in a human hepatocellular carcinoma cell line (HepG2) to suppress abnormal lipid metabolism [36].

Finally, NSAIDs express the ability to produce reactive oxygen species (ROS). For instance, celecoxib restricts cellular respiration and induces the extensive production of ROS during the apoptosis of murine metastatic cell lines (B16F10 and 4T1) [37]. In addition to the above, many anti-tumoral actions of NSAIDs were studied, and their specific mechanisms of action in multiple cancer cell lines are summarized below in Table 1.

## 4. Evidence of the Anti-Cancer Mechanisms of NSAIDs in In Vivo Models

The promising anti-cancer activities of NSAIDs were demonstrated in in vitro experiments, but additional successes were also reported in in vivo experiments. These successes could be divided into two major categories of NSAIDs applications, i.e., chemoprevention and chemotherapies. Chemoprevention capacity is brought by the anti-tumoral activity of NSAIDs in which the progression of cancer cell growth is diminished. The chemotherapeutic efficacy of NSAIDs is mainly based on their ability to prevent the metastatic spread of cancer cells and the inhibition of angiogenesis.

The administration of NSAIDs before cancer induction confirmed their chemopreventive capacities and abilities to significantly reduce the rate of tumor growth. In this area, the progression of colon tumors was effectively inhibited by piroxicam in a rat model [10]. In another study performed on the same model, Rao and co-workers also demonstrated that the application of sulindac inhibited the progression of invasive and non-invasive colon adenocarcinomas [7]. In the case of indomethacin, it was observed that its administration starting the same day of tumoral induction effectively reduced lung metastasis [52]. Furthermore, in C57BL/6 mice, indomethacin inhibited the progress of oral premalignant lesions in the squamous cell carcinomas of the head and neck [53].

Moreover, in chemotherapy, NSAID administration suppresses the metastatic spread of cancer cells. For example, in head and neck squamous carcinomas in C57BL/6 mice, indomethacin-induced immune cells suppressed the metastatic spread of cancer cells [53]. In addition, significant evidence has shown the tumoral volume reduction in adenocarcinomas [52,54,55]. For instance, celecoxib promoted apoptosis in vivo [56]. Furthermore, celecoxib decreased the invasion and metastasis of cancer cells in the PyMT/Col1a1 mice model of mammary tumors [57]. NSAIDs also downregulate angiogenesis in rodents. For instance, celecoxib inhibited angiogenesis in an ovarian cancer model of KpB mice [56].

Unfortunately, despite the promising results regarding chemoprevention and the reduction in neoplastic processes, in vivo assays of NSAIDs have also shown serious side effects. Adverse effects are the result of COX inhibition and refer to those associated with the gastrointestinal tract as well as additional problems associated with the kidney, liver, lung, bowel, and heart. Brown et al. reported that indomethacin and sulindac caused gastrointestinal side effects in Sprague-Dawley rats [58]. Additionally, piroxicam resulted in gastric ulceration and hepatic toxicity in Wistar albino male rats [59]. Moreover, the inhibition of COX-1 blocked the production of thromboxane. Comparatively, ketoprofen resulted in a higher blood bleeding time than non-selective and selective COX-2 inhibitors [60]. In the same way, they reduced the number of red blood cells and erythrocytes in albino NMRI mice [61]. In several studies, COX-2 selective inhibition did not have any reports of gastrointestinal toxicity [58,62]. In Sprague-Dawley rats, Brown et al. found that meloxicam and celecoxib did not cause any gastrointestinal side effects [58]. However, side effects appeared in other organs. In Dukrey rats, according to Niranjan et al., the oral administration of diclofenac and valdecoxib led to non-observable hemorrhagic leaks in the gastrointestinal tract but provided negative effects on the liver with regard to hepatitis and hepatic completion [62].

## 5. Evidence of the Anti-Cancer Mechanisms of Action of NSAIDs in Epidemiologic Studies

Numerous epidemiological studies have confirmed the chemopreventive and anti-cancer properties of NSAIDs, as described below, including the risk ratios (RRs), odds ratios (ORs), and standardized incidence ratios (SIRs) with 95% confidence intervals (CIs). A study among patients with large bowel cancers (1326 patients), other cancers (1011 patients), and patients without tumors (3880 patients) initially reported that previous histories of NSAIDs decreased the risk of developing human large bowel cancers (RR: 0.5; 95% CI: 0.4–0.8) [63]. Another study demonstrated that regardless of COX-1 and COX-2 inhibitors, a history of NSAID intake expressed chemopreventive potential in stomach cancer. Furthermore, Coogan et al. reported that among 254 stomach cancer patients, those who had taken NSAIDs for a minimum of four days per week for three months in the previous year before admission had a lowered risk of developing stomach cancer than non-NSAID users (OR: 0.3; 95% CI: 0.1–0.6). NSAIDs used by the above patients included acetylsalicylic acid, indomethacin, ibuprofen, mefenamic acid, and piroxicam [64]. Moreover, it was reported that the regular use of NSAIDs (non-selective COX inhibitors, COX-1 inhibitors, and COX-2 selective inhibitors) for six months during six years reduced the chances of peptic cancer in peptic ulcer patients from 27,016 non-NSAID users against 25,145 NSAID users (SIR: 0.79; 95% CI: 0.77–0.81) [65]. In an epidemiological study in colorectal cancer, acetylsalicylic acid users among 2279 colon cancer patients and 2907 non-colon cancer patients taking low dose acetylsalicylic acid at 75 mg for more than four tablets per week in more than a month had a lowered risk of developing colorectal cancer than non-acetylsalicylic acid users (OR: 0.78; 95% CI: 0.65–0.92) [66]. In addition, 17 years of data collected between 1994–2011 among 10,280 colorectal cancer patients and 102,800 non-cancer patients suggested that a minimum of five years of continued low acetylsalicylic acid (75–150 mg) intake reduced colorectal cancer risk by 27% (OR: 0.73; 95% CI: 0.54–0.99). The same study reported that the usage of COX-2 selective inhibitors reduced colorectal cancer risk (OR: 0.57; 95% CI: 0.44–0.74) [67]. Moreover, epidemiological evidence links the use of NSAIDs with metastasis prevention. In a meta-analysis, 202,780 patients with different types of cancers showed a reduction in distal metastasis during pre-diagnostic NSAID intake (RR: 0.708; 95% CI: 0.586–0.856) and post-diagnostic NSAID usage (RR: 0.708; 95% CI: 0.586–0.856) [68].

Even though epidemiological studies and clinical trials provide significant insight into the anti-tumoral activity of NSAIDs, several unwanted side effects of NSAIDs during long-term use have been reported. In a meta-analysis of 31 trials, including 116,429 patients subjected to cardiovascular risk assessments of NSAIDs (naproxen, ibuprofen, diclofenac, celecoxib, etoricoxib, rofecoxib, and lumiracoxib), it was suggested that rofecoxib was the most highly associated with cardiovascular risk (rate ratio: 2.12; 95% CI: 1.26–3.56) and ibuprofen was associated with a higher risk of stroke (rate ratio: 3.36; 95% CI: 1.00–11.6). In this study, even though all drugs showed an association with cardiovascular risk, diclofenac had the lowest risk among all selected NSAIDs (rate ratio: 3.98; 95% CI: 1.48–12.7) [69].

In addition, Bally et al. reported that 446,763 patients, including 61,460 individuals with acute myocardial infarctions, demonstrated that taking any dose of NSAIDs for up to a week increased the risk of myocardial infarction. In this study, the myocardial risk was observed in all NSAIDs, and the risk of myocardial infarction in decreasing order was rofecoxib (OR: 1.58; 95% CI: 1.07–2.17), naproxen (OR: 1.53; 95% CI: 1.07–2.33), diclofenac (OR: 1.5; 95% CI: 1.06–2.04), ibuprofen (OR: 1.48; 95% CI: 1.00–2.26), and celecoxib (OR: 1.24; 95% CI: 0.91–1.82) [70]. Moreover, Hamid et al. reported the induction of upper gastrointestinal complications with NSAIDs. Two years of data on 52 NSAID users suggested that the percentage of peptic cancer cases in acetylsalicylic acid and diclofenac users were associated with 30.7% and 32.7% of side effects, respectively. In this patient group, duodenal ulcers accounted for 65.3% of the cases, and gastric ulcers accounted for 42.3% [71]. In addition, a meta-analysis suggested that all NSAIDs increase the risk of upper gastrointestinal complications (rate ratio: 1.81; 95% CI: 1.17–2.81), especially diclofenac (rate ratio: 1.89; 95% CI: 1.16–3.09), ibuprofen (rate ratio: 3.97; 95% CI: 2.22–7.1), and naproxen (rate ratio: 4.22; 95% CI: 2.71–6.56) [72]. Moreover, NSAID use was also linked to acute kidney injury. Balestracci et al. reported in a one-year case study that ibuprofen was associated with 54% of acute gastroenteritis cases in children, as well as risk factors for acute kidney injury [73].

## 6. Combination of NSAIDs with Chemotherapeutic Drugs

Considering the reduced rate of newly available drugs, strategies intended to re-purpose currently marketed drugs as well as their combinations seem more feasible. Regarding the latter, it is possible to observe activity improvement depending on the synergistic effects of drug combinations. Therefore, for cancer therapy, combined chemotherapy was studied. Compared to monotherapy, combination therapy does not require high concentrations of drugs to obtain cytotoxic effects, thus reducing the adverse effects in healthy cells [74,75].

To date, some NSAIDs have been tested in combination with chemotherapeutic drugs (CTDs), immunotherapeutic agents, consumable products, other NSAIDs, and radiotherapies by undertaking in vitro, in vivo, and clinical studies for cancer therapeutics.

### 6.1. Combination of NSAIDs with Chemotherapeutic Drugs Studied in Tumoural Cell Lines

Chemotherapeutic drugs (CTDs) have the potential to downregulate the pathways associated with cancer, but in the case of some cancer cell lines, they are often not effective because of multidrug resistance (MDR). MDR is connected to complex changes in the cancer cellular environment, mainly caused by three factors, i.e., changes in hydrophilic drug transporters, higher energy requirements to influx hydrophobic cytotoxic drugs across the plasma membrane, and modifications in drug mechanisms of action [76]. Furthermore, hydrophobic drug resistance is associated with the ATP-binding cassette (ABC) transporter protein family, which is reported to efflux CTDs and lead to low drug accumulation in cells. In addition to the above, anti-apoptotic protein Bcl-2 overexpression was identified to be elevated during MDR [75].

Different proteins belonging to the ABC protein family restrict CTD transport. Firstly, p-glycoprotein (P-gp) is known to restrict daunorubicin accumulation [77]. Secondly, multidrug resistance-associated protein 1 (MRP1/ABCC1) restricts the entry of doxorubicin [78]. In addition, mitoxantrone resistance protein (MXR/ ABCG2) and breast cancer resistance protein (BCRP) restrict mitoxantrone intake [79,80].

NSAIDs and CTDs were combined to examine the potential of NSAIDs for chemosensitization. The application of CTDs alone in tumoral cell lines initially boosts the downregulation of cell proliferation. However, after a certain period of continued therapy, the drug concentration is reduced, and CTDs are expelled out of the cells. This may be caused by the MDR expression caused by CTDs. In this area, it was proven that doxorubicin treatment for several days had the potential to cause MDR-1 gene expression, leading to P-gp surface glycoproteins in tumoral cells. The application of NSAIDs in combination negatively affected both P-gp accumulation and MDR-1 gene expression. NSAIDs expressed additive potential to multiple CTDs, as Duffy and co-workers reported. They found that indomethacin and sulindac exerted synergic effects with anthracyclines (doxorubicin, daunorubicin, and epirubicin), vincristine, teniposide, and VP-16 [74].

Additionally, Arunasree and co-workers reported that the combination of celecoxib with doxorubicin, vincristine, etoposide, and irinotecan provided synergic effects in two different neuroblastoma cell lines, SH-SY5Y and SK-N-BE [75]. COX-2 selective inhibitors provide additive effects in combination with CTDs and are considered to downregulate P-gp via COX-2 inhibition [75,81]. The non-cytotoxic concentration of COX-2 selective inhibitors, celecoxib, and NS-398, downregulated both P-gp and COX-2 with a significant correlation. Even though the inhibition of P-gp is concentration-dependent, the low cytotoxicity of COX-2 inhibitors has less potency to downregulate COX-2 expression. In addition to the above, there are two reported pieces of evidence to consider for the COX-2 independent mechanism. Firstly, the non-COX inhibitor sulindac sulphone in combination with CTDs downregulated tumoral cell proliferation, and secondly, Xia and co-workers reported that celecoxib at a non-cytotoxic concentration of 50 µm inhibited the expression of P-gp in MCF-7 and JAR/VP16 cell lines [74,82].

**Figure 2:**
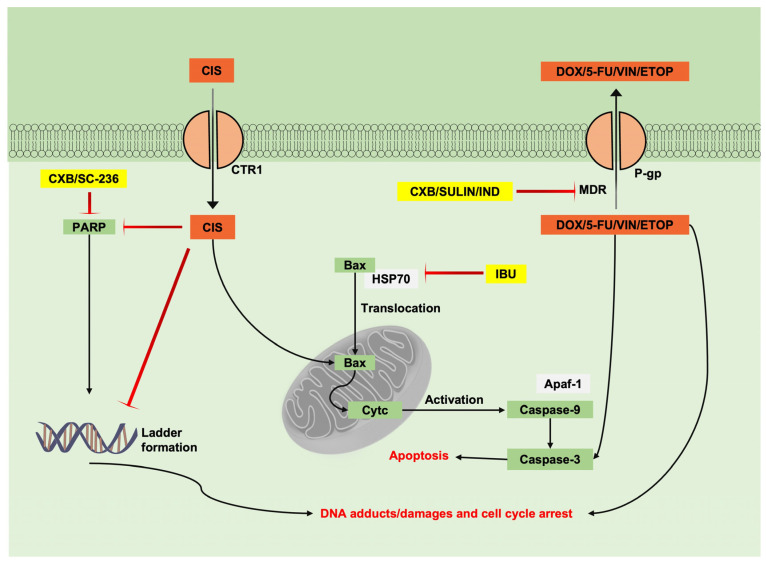
In vitro evidence of the combined mechanisms of NSAIDs and chemotherapeutic agents.

As shown in Figure 2, in MDR, P-gp expels CTDs out of the cells, resulting in the low drug accumulation of doxorubicin, 5-fluorouracil, vincristine, and etoposide. However, it is possible to assume that NSAIDs, such as celecoxib, sulindac, and indomethacin, inhibit P-gp expression from facilitating CTD action. A combination of celecoxib and doxorubicin resulted in cell cycle arrest at the G1 phase of colon tumoral cells (HCT116), and in combination with ibuprofen and cisplatin in lung adenocarcinoma cells (A549), ibuprofen demonstrated to translocate the Bax protein to mitochondria by inhibiting heat shock protein 70 (Hsp70), facilitating cisplatin-mediated Bax activation, leading to Apaf-1 activation followed by the activation of cytochrome-c and the phosphorylation of caspase-9, resulting in the apoptotic cell death of A549 cells. In the combination of cisplatin and ibuprofen, cisplatin and other platinum-based drugs are transported into tumoral cells by the copper transporter 1 (CTR1) protein. Thus, the antagonistic nature of ibuprofen in inhibiting the CTR1 transporter pump is unknown. Further findings elucidated the common potentials of NSAIDs and CTDs individually to interfere with cancer mechanisms. COX-2 selective inhibitors celecoxib and SC-236, along with CTDs cisplatin and etoposide, caused DNA adducts and poly (ADP-ribose) polymerase (PARP) inhibition. Moreover, a common ability to activate caspase-3 and caspase-9 by non-COX selective inhibitor ibuprofen and CTDs, such as cisplatin and 5-fluorouracil, was also proven [83,84,85]. Moreover, doxorubicin metabolites share the potential to activate free oxygen radicals from ROS during mitochondrial deletion to cause apoptotic cell death.

Despite the chemosensitizing potentials of NSAIDs being independent of COX inhibition, NSAIDs, such as indomethacin and sulindac, are less effective when combined with 5-fluorouracil, methotrexate, cyclophosphamide, cytarabine, and hydroxyurea [74]. Certain NSAIDs, such as celecoxib and SC-236, possess antagonistic effects that combine with platinum-based compounds. A similar finding demonstrated that administering celecoxib in human gastric tumoral cells caused antagonistic effects in combination with cisplatin. However, the resulting antagonistic behavior of NSAIDs on platinum-based drugs had no connection to COX and PGE_2_ inhibition [85]. NSAIDs lower the intracellular accumulation of platinum-based drugs. Platinum-based drugs are polar and transported via plasma membrane transporter proteins. In this area, Chen and co-workers reported that a combination of celecoxib and cisplatin reduced cisplatin intracellular accumulation and both decreased CTR1 protein expression [85].

A novel strategy to improve drug permeability is the conjugation of CTDs with NSAIDs. It has several advantages, such as pharmaceutical activity improvement and the increased lipophilicity of CTD. NSAID-CTD conjugates, such as acetylsalicylic acid-cisplatin, ketoprofen-cisplatin, and naproxen-cisplatin, improved cisplatin permeability and were found to enhance cisplatin’s effects [86].

### 6.2. Combination of NSAIDs with Chemotherapeutic Drugs in In Vivo Models

In in vitro models, combinations of NSAIDs and cytostatics seem more effective in overcoming MDR than in in vivo models. There is only a little evidence regarding NSAIDs’ potential in combination with CTDs for MDR-1 and P-gp inhibition [87,88].

CTDs, such as doxorubicin, exerted synergic pharmacological actions with celecoxib, diclofenac, and sulindac and were identified to inhibit pathways relating to MDR. Individually, a combination of sulindac and doxorubicin delayed the MDR-1 expressing xenograft of human large-cell lung carcinoma (NCI H460) in mice. It is one of the first pieces of evidence where sulindac blocks the efflux of doxorubicin by MDR-1-expressing carcinoma cells [89]. Moreover, Awara and co-workers assessed celecoxib and diclofenac in combination with doxorubicin in Ehrlich carcinoma cells and reported P-gp inhibition by both NSAIDs, resulting in improved doxorubicin intake [87]. Further, similar to the in vitro evidence, the combination of celecoxib and 5-fluorouracil improved the expression of cytochrome-c, caspase-3, and caspase-9 in xenografts of human colon cancer cells (HT-29) [88].

In the in vivo models, the major endpoints were related to the increased activity of the combination of NSAIDs with CTDs toward tumoral growth delay, angiogenesis, and anti-metastasis. In a wide range of in vivo studies, apoptosis was not obtained by free CTDs, free NSAIDs, or their combinations [88,89,90,91]. The combination of NSAIDs and CTDs only delayed tumoral growth. In contrast, Zhang and co-workers reported an increase in apoptotic cells using the combination of celecoxib and 5-fluorouracil [88]. Thus, combining NSAIDs delayed tumoral growth in comparison to monotherapy. Moreover, O’Connor and co-workers reported that the administration of doxorubicin or sulindac had no significant tumor delay, but their combination significantly delayed tumor growth in xenografts of human lung cancer [89]. Tumoral cell proliferation and volume are influenced by angiogenesis, which is characterized by the elevation of microvascular density. A study regarding drug combinations’ influence on microvascular density identified that celecoxib, in combination with doxorubicin, declined microvascular density. However, their combination with irinotecan had no effect on the microvascular density of the neuroblastoma xenografts of SH-SY5Y cells [90]. Unlike the in vitro evidence, sulindac and indomethacin possessed synergic effects with cisplatin and caused tumoral growth delays. However, cisplatin withdrawal and NSAIDs’ influence on CTR1 is unknown in vivo. Furthermore, the induction of chemotaxis as a result of chemotherapy ignited the search for the anti-metastasis activity of NSAIDs. The post-administration of NSAIDs after chemotherapy has proven to prevent chemotaxis. For instance, Gunjal and co-workers reported the influence of ibuprofen in preventing the metastasis caused by cisplatin post-chemotherapy in xenografts of human ovarian cancer (A2780) cells [92].

### 6.3. Combination of NSAIDs with Chemotherapeutic Drugs Studied in Clinical Trials

Pre-clinical data has suggested that a combination of CTDs and NSAIDs significantly impacts the progression of cancer. Currently, several clinical trials combining NSAIDs and CTDs are being undertaken. Most of the clinical trials are in phase one or two and are subject to evaluations for safety and clinical significance [93,94,95,96]. The mixture of rofecoxib with cyclophosphamide and vinblastine in a phase two trial among advanced solid tumor patients exhibited a 30% clinical benefit [93]. However, rofecoxib was withdrawn in 2004 after its use was associated with an instance of myocardial infarction [97].

The immunohistochemical elevation of COX-2 was considered in a wide range of clinical studies to target a combination of CTDs with selective COX-2 inhibitors in chemotherapy. Despite COX-2 selective inhibition, celecoxib in a phase two randomized clinical trial on peritoneal carcinoma showed no significant COX-2 inhibition [94]. In several studies, celecoxib administration had a low impact on anti-angiogenic and anti-metastatic features in combination with carboplatin, and no changes were observed in the VEGF and endostatin serum levels [94,96]. In contrast, celecoxib with cyclophosphamide caused a decreased serum VEGF level that demonstrated anti-angiogenesis potential in metastatic breast cancer [95]. Similar to the pre-clinical evidence, the oral administration of celecoxib improved a greater number of responses in patients with platinum-based drug resistance [96] and was recently reported to lower doxorubicin-induced MDR by decreasing P-gp expression in patients’ biopsy samples of canine lymphoma [98].

Celecoxib, in combination with cisplatin, irinotecan, and carboplatin, was administered via an oral dose of 400 mg per day, and several side effects were reported [96,99]. The chemotherapeutic side effects included diarrhea, rectal bleeding, and abdominal pain. Even though the addition of NSAIDs along with CTDs is beneficial to extend the average patient survival rate, it is crucial to also consider the side effects. Among the phase one and two clinical trials, celecoxib expressed mild cases of febrile neutropenia, sepsis, severe dehydration, nausea, and diarrhea in the case of patients with esophagus cancer [100], whereas in ovarian cancer, patients expressed side effects such as gastroduodenal perforations and intestinal bleeding [96]. In the case of epithelial ovarian cancer, mild skin reactions and cardiovascular morbidity were observed [94]. Combination chemotherapy is still used and exhibits results, although the number of patients that stop the treatment due to side effects is also high.

## 7. Combination of NSAIDs with Phosphatidylcholine

Phospholipids form the bilayer of all cell membranes. In cells, the most abundant phospholipids are phosphatidylcholine (PC) and phosphatidylethanolamine (PE) at approximately 50% and 30%, respectively. PC is the main class of phospholipids in the cell membrane of eukaryotic cells, and cellular organelles, such as the endoplasmic reticulum, Golgi body, mitochondria, lysosome, and nucleus, contain PC [101]. PC is a glycerophospholipid containing a head group, choline linked to a phosphate group, an apolar group, and two fatty acid chains at the *sn-1* and *sn-2* positions of the glycerol skeleton.

Since the 1980s, the Lichtenberger research group has been a pioneer in the studies of the influence and importance of PC on gastric protection after the administration of NSAIDs [102]. The evaluation trials of NSAIDs’ influence on gastric PC were initiated in 1983 [102] and showed that the intraluminal administration of surface-active phospholipids effectively protected the gastric mucosa from acid-induced necrosis and bleeding. They also demonstrated that the concentration of surface-active phospholipids in the gastric mucosa is markedly increased via treatment with prostaglandins [102]. It was proven that a reduction in the surface hydrophobicity of the stomach caused by the administration of aspirin could be completely reversed by the addition of 16,16-dimethyl prostaglandin E_2_ [103].

The gastric epithelium produces bicarbonate ions which are entrapped by glycoproteins in the mucosal barrier, which generates a gradient pH range across the mucosal layer. The negatively charged and hydrophilic gastric mucus barriers are attached to a positively charged head group of PC, forming a hydrophobic coating that protects the gastric epithelium against acidic pH conditions [104].

NSAIDs are acidic and attach to the head group of PC via an ionic bond. For instance, dipalmitoylphosphatidylcholine, a zwitterionic PC in the gastric mucus, interacts with NSAIDs, such as acetylsalicylic acid, indomethacin, diclofenac, and naproxen, in an acidic medium [105].

Concerns regarding the application of NSAIDs and gastrointestinal toxicity started to be reduced after the first attempts to combine NSAIDs and PC. One of the first successful clinical demonstrations of the reduction in gastrointestinal toxicity was achieved via the application of equal rations of neutralized acetylsalicylic acid and PC [106]. In further clinical studies, it was also proven that a mixture of ibuprofen and PC did not result in the side effects associated with gastrointestinal toxicity, and the drug had the same bioavailability after application in the free form as in the mixture [107].

A homogenized mixture of indomethacin and PC was also evaluated in a mice model for gastrointestinal safety, where side effects caused by indomethacin monotherapy were reduced in a combined treatment [108]. A similar observation was reported in a clinical trial for the combination of acetylsalicylic acid and PC, which reduced acetylsalicylic acid-induced gastroduodenal erosion and ulceration in human subjects [109]. Thus, a mouse model expressed equal inhibition of synovial fluid PGE_2_ expression in the treatment of indomethacin monotherapy and combination with PC and ensured gastrointestinal safety [108].

More interestingly, in a recent report, the NSAID and PC combination was identified to have anti-tumoral activity in cancer cell lines and mice models, and, in mice, combinations of indomethacin and acetylsalicylic acid with PC proved gastrointestinal safety and prevented the metastatic spread of cancer cells [110]. Comparatively, the anti-tumoral efficacy of the combination improved from NSAID monotherapy. In several in vivo studies, acetylsalicylic acid, indomethacin, and sulindac combined with PC expressed anti-tumoral activity against colon cancer in mice models [110,111].

**Figure 3:**
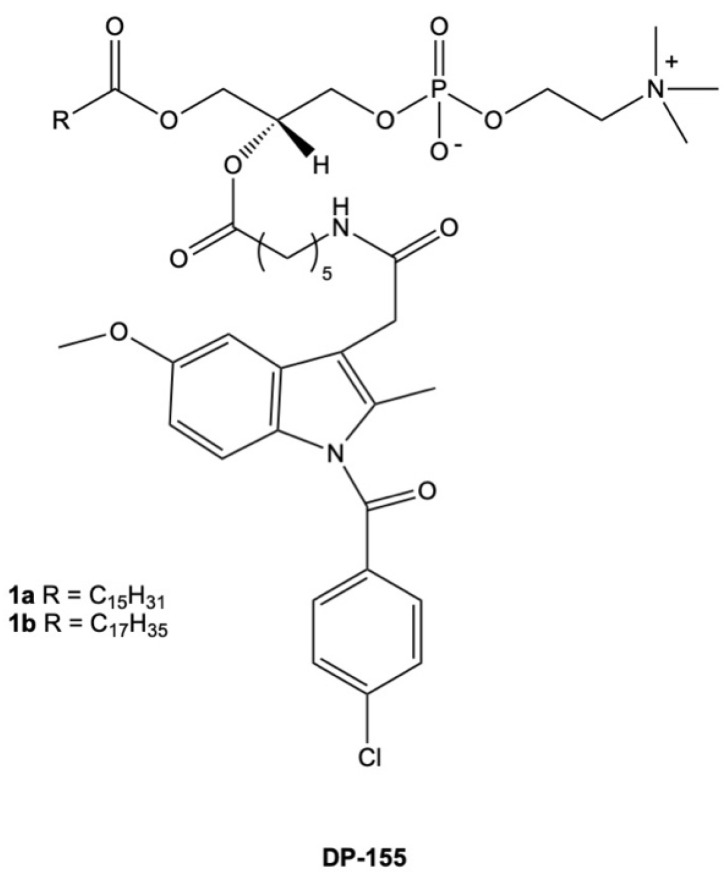
Chemical structure of conjugates of phosphatidylcholine with indomethacin (DP-155).

A novel strategy for the delivery of NSAIDs and phospholipids is based on their direct attachment via covalent bonds between the drug and the skeleton of phospholipids. This type of conjugate was obtained for indomethacin. Hybrid DP-155 (Figure 3) was a mixture of phosphatidylcholines **1a** and **1b,** which contained palmitic and stearic acids in the *sn-1* position and a drug attached to the *sn-2* position of PC through a 5-carbon linker. DP-155 was also synthesized by D-Pharm LTD as a potential agent active in Alzheimer’s disease. DP-155 lowered gastrointestinal and renal toxicity by 10 and 5-fold, respectively, than free indomethacin [112]. Moreover, it was confirmed that the conjugates of indomethacin with PC had lower concentrations in plasma in comparison with the free drug but had a much higher relative concentration in the brain.

**Figure 4:**
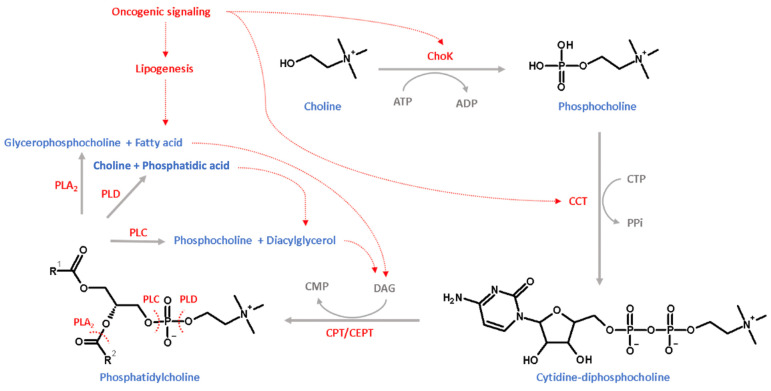
Targets of oncogenic signaling in the CDP-pathway.

During cancer, cell proliferation, the cell cycle, and survival cellular metabolism are reprogrammed to increase biomass [113]. PC synthesis plays a crucial role in the biomass extension of tumoral cells [113,114]. In 1950, Kennedy and co-workers established the PC synthesis pathway, named the Kennedy pathway or cytidine-choline (CDP-choline) pathway [115]. As shown in Figure 4, in the CDP-choline pathway, PC and its intermediates are governed by secondary signals in the oncogenic mechanism. PC synthesis in the CDP-choline pathway is ATP-dependent and initiated by the intake of choline with sodium-dependent choline transporter-1 (CHT1), sodium-dependent choline transporter-like protein (CTL), and organic cation transporters [116]. Further, it is phosphorylated to phosphocholine by ATP-dependent choline kinase (ChoK), and the nucleotide cytidine is attached to phosphocholine to form cytidine-diphosphocholine via the catalyzation of cytidylyltransferase (CCT). Finally, PC is synthesized from the transformation of the phosphocholine moiety to diacylglycerol (DAG) via the catalyzation of choline/ethanolamine phosphotransferase (CPT/CEPT) [114]. In addition, as shown in Figure 4, PC is catabolized by phospholipase A_2_ (PLA_2_) to glycerophosphocholine and fatty acids and catabolized by phospholipase C (PLC) to phosphocholine and DAG and also catabolized by phospholipase D (PLD) to choline and phosphatidic acid. Considering the transformation of the tumoral cellular mechanism, several studies reported evidence of accelerated PC synthesis and the improved activity of phospholipases. Tumoral intracellular metabolism promotes the intake of choline by enhancing the expression of CHT1 and CTL3 in esophageal, colon, breast, prostate, and ovarian tumoral cells [117,118,119]. In addition, ChoK is not the key limiting step in the CDP-choline pathway, although ChoK plays a comprehensive step in activating PC synthesis from oncogenic signaling pathways. In addition, c-MYC regulates the CDP-choline pathway by regulating CCT expression [120]. In several studies, the inhibition of ChoK significantly induced PC production and was considered a suitable strategy to inhibit the tumoral cell cycle [121,122]. In addition, García-Molina and co-workers reported that in mammary and liver tumoral cell lines, the inhibition of ChoK expression suppresses tumoral cell proliferation [123]. In comparison to normal cells, tumoral cell PC metabolism enhances choline product transport and PC-specific phospholipases [124].

**Figure 5:**
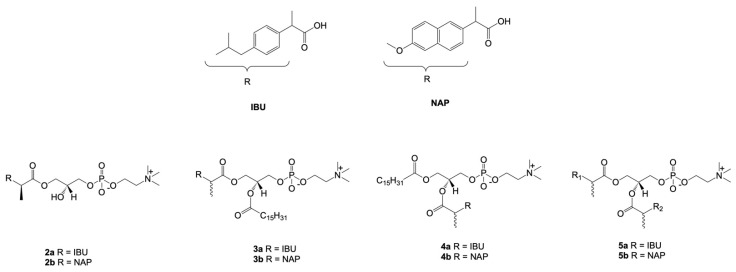
Chemical structures of the conjugates of phosphatidylcholine with ibuprofen and naproxen tested for anti-cancer activity against cancer cell lines.

In the face of the above-mentioned facts, the synthesized conjugates of phosphatidylcholine with naproxen (NAP) and ibuprofen (IBU) (Figure 5) were also evaluated in human promyelocytic leukemia cells (HL-60), human colon cancer cells (Caco-2), and human non-tumorigenic intestinal epithelial cells (IPEC-J2) [125]. Therapeutic concentrations of IBU and NAP in HL-60 and Caco-2 were in a range from 78.63 to 140.51 µM. However, the incorporation of these drugs in the *sn*-1 position of lysophosphatidylcholine (LPC) (**2a**, **2b**) did not produce derivatives with higher activities. Despite this, the conjugation of PC with drugs in both or only one position produced active biomolecules. Phospholipids containing palmitic acid (PA) in the *sn-2* position and IBU or NAP in the *sn-1* position exhibited higher cytotoxic effects and expressed no activity in the normal cell line, IPEC-J2. The most active derivative in this group was compound **3b,** with an IC_50_ value of 27.13 µM. In contrast, disubstituted PC **5a** and **5b** expressed almost two times higher activity in comparison to the free drugs.

## 8. Combination of NSAIDs with Terpenoids

Terpenoids are secondary metabolites in plants and animals. This large class of naturally occurring substances has been extensively studied for decades for their anti-inflammatory, anti-cancer, anti-bacterial, anti-fungal, anti-viral, and anti-parasitic capacity [126]. A vast number of unmodified terpenoids were tested for their anti-cancer activity. Despite this, due to their rapid metabolism and low bioavailability, terpenoids have not been clinically applied for cancer [127].

The high potential of terpenoids and the mentioned obstacles for their industrial applications as anti-cancer agents were the basis for studying a new concept based on the molecular hybridization (MH) of terpenoids with well-known anti-inflammatory drugs indicated in the literature as useful in anti-cancer therapy. Combinations of NSAIDs and terpenoids provide several beneficial effects, such as bioavailability, solubility, protection from toxicity, and permeation. The effectiveness of novel codrugs containing terpenoids and NSAIDs as pharmacophoric units was tested using three different strategies. An initial approximation was carried out by preparing physical mixtures of NSAIDs and terpenoids; a second strategy consisted of using terpenoids as deep eutectic solvents (DESs) to carrier NSAIDs, and the third methodology was the conjugation of terpenoids and NSAIDs. Each preparation enhanced the application of both groups of compounds.

In physical mixtures, terpenoids improved the skin permeation of NSAIDs. In particular, the transdermal permeation of indomethacin and tiaprofenic acid was enhanced during formulation with menthol [128,129]. Several terpenes, such as limonene, menthol, and nerolidol, are known to disrupt the intracellular lipids of the stratum corneum, which eventually enhances the transdermal delivery of NSAIDs and reduces direct gastrointestinal contact [129].

The application of terpenes in therapeutic deep eutectic systems (THEDESs) is based on the preparation of a binary mixture of terpenes and drugs in different molar ratios. As a result, it is possible to improve the bioavailability and solubility of NSAIDs. In recent decades, several monoterpenes, namely limonene (LIM), menthol (ME), perillyl alcohol (POH), and thymol (THY), were used for the preparation of THEDESs with NSAIDs. On the contrary, due to the well-assessed anti-cancer activity and low solubility, the only studied NSAID for this type of formulation with anti-cancer activity was IBU [127,130,131]. As provided in Table 2, the formulated THEDESs, compared to their individual compounds, showed higher selectivity in cancer cells than in normal cells. The LIM: IBU (4:1) formulation inhibited the proliferation of HT29 cells without disrupting cell viability. In addition, the mechanism of action was different from the individual compounds used in the preparation of THEDESs [127]. The first strategy of the preparation of terpenes and NSAIDs enhanced skin permeation. In contrast, a physical mixture of terpenes and NSAIDs was less effective in cancer cell lines. Thus, the same ratio of compounds in a THEDES was twice effective in improving the antiproliferative activity. The EC_50_ value of the POH: IBU (3:1) hybrid was much lower than the EC_50_ of the mixture of POH and IBU (3:1) (Table 2) [130]. In addition to the anti-cancer activity, the THEDES demonstrated improved solubility in a PBS buffer. Among the similar molar ratios of the THY: IBU (3:1) and ME: IBU (3:1) formulations and their physical mixtures, comparatively, ME: IBU (3:1) had higher solubility in phosphate saline buffer, but THY: IBU (3:1) was more cytotoxic in Caco-2 cells (Table 2) [131].

**Figure 6:**
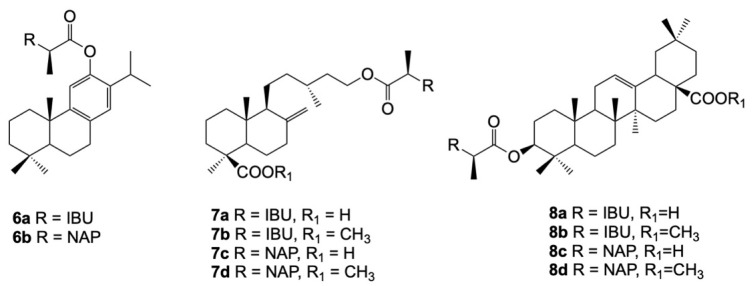
Chemical structures of terpenes conjugated with ibuprofen and naproxen, assessed for their anti-cancer activity.

The preparation of the hybrid molecules of the NSAIDs and natural terpenes is a new approach focused on improving anti-inflammatory activity. In this aspect, diterpenes and triterpenes were conjugated with ibuprofen (IBU) or naproxen (NAP) (Figure 6). Ferruginol, imbricatolic acid, and oleanolic acid, via their free hydroxy groups, were esterified with the carboxylic group of the selected drugs. The topical anti-inflammatory effects of the synthesized hybrids (**6a–b**, **7a–d**, **8a–d**) were evaluated in mice, lung fibroblasts (MTC-5), gastric epithelial AGS cells, and hepatocytes (HepG2) [132]. The studied conjugates showed a remarkable increase in activity in comparison with the free terpenes and free drugs.

Ferruginyl ibuprofenate (**6a**) and ferruginyl naproxenate (**6b**) showed less cytotoxic activity against the three studied cell lines and better anti-inflammatory effects than their parent diterpene. Among the imbricatolic acid derivatives, hybrid **7a** and **7c** were more active as cytotoxic agents than their starting molecules before conjugation. It also turned out that the small difference in the presence of the methyl ester of the carboxylic group changed the hybrid compound’s anti-inflammatory and cytotoxic activities. After the methylation of the free COOH groups, the IC_50_ values of **7b** and **7d** were higher and ranged from 601 to more than 1000 μM [132]. However, methylation provided better anti-inflammatory activity and lower cytotoxicity to this type of derivate [133]. Oleanolic acid was selected for conjugation with multiple NSAIDs due to its selectivity in anti-inflammatory action [132,134]. Among their hybrids with NSAIDs, molecules **8a–d** exhibited less cytotoxicity in the studied cell lines. Due to their larger molecular size, rather than binding to COX-2 enzymes, they block their substrates and provide better anti-inflammatory activity. In in vivo models, **8a**, **8b**, and **8d** showed especially higher anti-inflammatory activities than other synthesized hybrid compounds [132].

**Figure 7:**
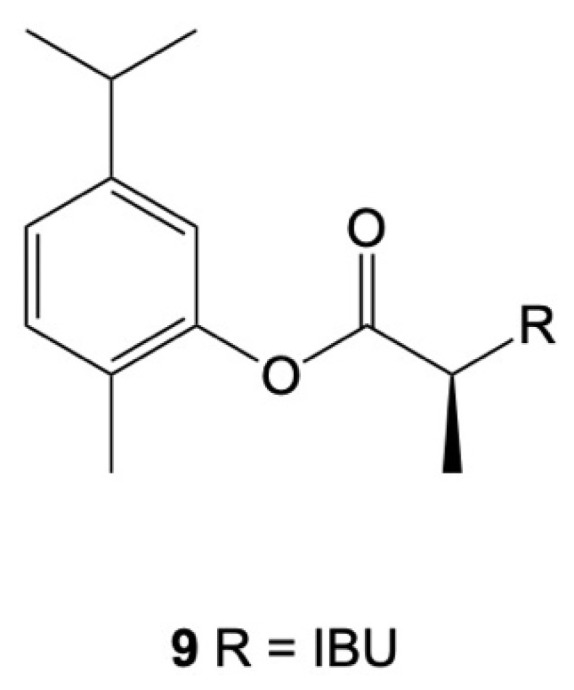
Chemical structure of CVIB.

Rolim and co-workers synthesized CVIB, a codrug developed via the association of carvacrol (a phenolic terpene) and IBU (Figure 7). The chemical bond between the two active pharmacophores provided a new molecule with suitable enzymatic stability, promoting improvements in the anti-inflammatory properties in the in vitro and in vivo models. CVIB was able to reduce inflammation and leukocyte migration as well as the production of inflammatory mediators. Its anti-inflammatory potential and bioavailability in human plasma highlighted that this type of conjugation constitutes a better approach than the physical mixture of the parent compounds [134].

## 9. Conclusions

This review highlights the anti-tumoral potential of NSAIDs. Evidence of the mechanisms of NSAIDs in monotherapies and in combination with CTDs was discussed at different levels, including in tumoral cell culture, animal models, and clinical trials. In brief, experiments carried out in this area mainly consider exploring COX-dependent and COX-independent targets of NSAIDs involved in the inhibition of cell proliferation, metastasis, neoplasia, and angiogenesis. Even though the combinations of NSAIDs and CTDs were reported, many side effects were also described. Among them, gastrointestinal toxicity was the most relevant.

Therefore, the literature’s data indicates that an effective method to overcome the toxicity of NSAIDs to use them as anti-cancer and anti-inflammatory compounds is their mixing or hybridization with natural compounds, such as phospholipids and terpenoids. In particular, hybrid compounds present superior therapeutic efficacies than physical mixtures. Current evidence shows that mixtures of NSAIDs and PC and their hybrids not only decrease side effects but also improve therapeutic anti-cancer activities. Therefore, it could be assumed that, in the future, this research line needs to be better further investigated. Special attention should be paid to the design and development of methods for obtaining highly active conjugates whose anti-cancer effects could be then enhanced using nanotechnology, which is known as a promising tool for the safe and effective delivery of NSAIDs.

## Figures and Tables

**Table 1:** Anti-tumoral mechanisms of action of NSAIDs in in vitro studies.

NSAIDs	Therapeutic Concentration	Cancer Cell Line	Mechanism of Action	Reference
Acetylsalicylic acid	0.5–4 mM for 2.5 h	MDA-MB-231, B16F10, CHO K1, and U87-MG	Dose-dependent direct binding on an oncogenic extracellular matrix enzyme called heparinase and the inhibition of cell migration and angiogenesis.	[38]
	0.1–1 mM for 12 h	SW480	Dose-dependent relocation of EGF and the phosphorylation of EGFR.	[39]
Indomethacin	0.4 mM for 48 h	EC109	Induction of mitochondria-derived caspase-3 in apoptosis.	[40]
	0.1–0.3 mM for 24 h	A375, MeWo, and SK-MEL-5	Dose-dependent downregulation of survival via ROS induction and NFκB signaling. Induced ROS upregulation of DR5 and CHOPS in TRAIL-related cell death.	[41]
Diclofenac	0.1 and 0.2 mM for 24 h	4T1	Downregulation of lactate secretion in T-cell-mediated cell death.	[42]
	0.4 and 0.8 mM for 48 h	MDA-MB-231 and HCC1937	Dose-dependent downregulation of GLUT1 and c-Myc expression, the inhibition of hexokinase activity, and the inhibition of cell proliferation.	[43]
Ibuprofen	0.5 mM for 48 h	AGS	Downregulation of VEGF-A, PCNA, Akt, CD44, and OCT3/4 gene transcription in apoptosis.	[28]
	2 mM for 24 h	HTZ-349 and A172	Downregulation of c-Myc expression in the inhibition of cell growth and migration.	[44]
Naproxen	0.5–2 mM for 72 h	UM-UC-5 and UM-UC-14	Dose-dependent downregulation of Bcl-2 and the upregulation of Bax expression during apoptosis.	[30]
	6 mM for 6 h	MDA-MB-231	Increased activation of caspase-3 and caspase-9 in apoptosis.	[45]
Sulindac	0.03–0.12 mM for 24 h	HCT116	Dose-dependent induction of ER stress makers, such as DR5, pPERK, and pEIF2α in ER-mediated apoptosis.	[46]
	0.03–1 mM for 48 h	FaDu	Induction of the production of VEGFR–2 and the arrest of cells at the G2/M phase.	[47]
Piroxicam	0.025–0.05 mM for 24 h and 48 h	MCF-7 and MDA-MB-231	Time-dependent downregulation of IL-1*β* and IL-6 gene expression.	[48]
	0.03 mM for 3–48 h	MCF-7	Time-dependent induction of ROS-activated PI3K/Akt signaling during apoptosis.	[49]
Celecoxib	0.02–0.04 mM for 24 h	A375 and Mel-STM	Inhibition of COX-2 expression and the downregulation of cell migration.	[50]
	0.04, 0.08 and 0.1 mM for 24–48 h	MDA-MB-231 and SK-BR-3	Dose-dependent and time-dependent upregulation of caspase-3 induced cell cycle arrest at the G1 and G2 phases.	[51]

**Table 2:** EC_50_ values of the cytotoxicity and antiproliferative assays of the THEDESs and compounds. Cytotoxicity assays were carried out on the human epithelial colorectal adenocarcinoma cell line (Caco-2). The antiproliferative activity was assessed using the human Caucasian colon adenocarcinoma cell line (HT29).

Compound/THEDES	Molar Ratio	EC_50_ Values (mM)		Selectivity Index
		Colon Adenocarcinoma Cell Line (HT29)	Cytotoxicity Assay	
IBU	-	2.346 ± 0.09	2.89 ± 0.06	1.23
LIM	-	0.67 ± 0.03	2.64 ± 0.11	-
POH	-	2.37 ± 0.20	4.86 ± 1.58	2.06
THY	-	5.22 ± 1.16	6.73 ± 1.69	1.29
ME	-	4.31 ± 0.63	5.09 ± 0.73	1.18
POH + IBU	3:1	4.51 ± 0.26	-	-
LIM:IBU	4:1	2.390 ± 2.919	10.5 ± 0.883	-
POH:IBU	3:1	1.316 ± 0.07	8.46 ± 1.13	5.89
THY:IBU	3:1	0.30 ± 0.04	1.07 ± 0.37	3.5
ME:IBU	3:1	4.3 ± 0.71	8.92 ± 1.39	2.07

## Abbreviations

PLA_2_	Phospholipase A_2_
Tr-NSAID	Traditional NSAIDs
TX	Thromboxane
EGFR	Epidermal growth factor receptor
Caspase	Cysteine-aspartic proteases-
DR5	Death receptor 5
CHOPS	CCAAT/enhancer binding protein homologous protein
TRAIL	Tumor necrosis factor related apoptosis inducing ligand
GLUT1	Glucose transporter-1
STAT-3	Signal Transducer and Activator of Transcription 3
ER	Endoplasmic reticulum
pEIF2α	Phosphorylated eukaryotic initiation factor 2 α
VEGFR-2	Vascular endothelial growth factor receptor–2
IL	Interleukin
PARP	Poly (ADP-ribose) polymerase
HSP70	Heat shock protein 70
Cyt-c	Cytochrome-c
Apaf-1	Apoptotic protease activator 1
CXB	Celecoxib
CIS	Cisplatin
IBU	Ibuprofen
SULIN	Sulindac
IND	Indomethacin
DOX	Doxorubicin
5-FU	5-Florouracil
VIN	Vincristine
ETOP	Etoposide
PLC	Phospholipase C
